# Maternal Human Milk Oligosaccharide Profile Modulates the Impact of an Intervention with Iron and Galacto-Oligosaccharides in Kenyan Infants

**DOI:** 10.3390/nu11112596

**Published:** 2019-10-29

**Authors:** Daniela Paganini, Mary A. Uyoga, Guus A.M. Kortman, Jos Boekhorst, Sacha Schneeberger, Simon Karanja, Thierry Hennet, Michael B. Zimmermann

**Affiliations:** 1 Laboratory of Human Nutrition, Department of Health Sciences and Technology, ETH Zurich, 8092 Zurich, Switzerland; mary.uyoga@hest.ethz.ch (M.A.U.); michael.zimmermann@hest.ethz.ch (M.B.Z.); 2 Department of Medical Epidemiology, College of Health Sciences, Jomo Kenyatta University of Agriculture and Technology, 00200 Nairobi, Kenya; skaranja@jkuat.ac.ke; 3 NIZO Food Research BV, 6718 ZB Ede, The Netherlands; Guus.Kortman@nizo.com (G.A.M.K.); Jos.Boekhorst@nizo.com (J.B.); 4 Department of Physiology and Zurich Center for Integrative Human Physiology, University of Zurich, 8057 Zurich, Switzerland; sacha.schneeberger@uzh.ch (S.S.); thierry.hennet@uzh.ch (T.H.)

**Keywords:** human milk oligosaccharides, infant, iron, Africa, Kenya, prebiotic, galacto-oligosaccharides, gut microbiota, secretor, micronutrient powder

## Abstract

There is little data on human milk oligosaccharide (HMO) composition in Sub-Saharan Africa. Iron fortificants adversely affect the infant gut microbiota, while co-provision of prebiotic galacto-oligosaccharides (GOS) mitigates most of the adverse effects. Whether variations in maternal HMO profile can influence the infant response to iron and/or GOS fortificants is unknown. The aim of this study was to determine HMO profiles and the secretor/non-secretor phenotype of lactating Kenyan mothers and investigate their effects on the maternal and infant gut microbiota, and on the infant response to a fortification intervention with 5 mg iron (2.5 mg as sodium iron ethylenediaminetetraacetate and 2.5 mg as ferrous fumarate) and 7.5 g GOS. We studied mother–infant pairs (*n* = 80) participating in a 4-month intervention trial in which the infants (aged 6.5–9.5 months) received daily a micronutrient powder without iron, with iron or with iron and GOS. We assessed: (1) maternal secretor status and HMO composition; (2) effects of secretor status on the maternal and infant gut microbiota in a cross-sectional analysis at baseline of the intervention trial; and (3) interactions between secretor status and intervention groups during the intervention trial on the infant gut microbiota, gut inflammation, iron status, growth and infectious morbidity. Secretor prevalence was 72% and HMOs differed between secretors and non-secretors and over time of lactation. Secretor status did not predict the baseline composition of the maternal and infant gut microbiota. There was a secretor-status-by-intervention-group interaction on *Bifidobacterium* (*p* = 0.021), Z-scores for length-for-age (*p* = 0.022) and weight-for-age (*p* = 0.018), and soluble transferrin receptor (*p* = 0.041). In the no iron group, longitudinal prevalence of diarrhea was higher among infants of non-secretors (23.8%) than of secretors (10.4%) (*p* = 0.001). In conclusion, HMO profile may modulate the infant gut microbiota response to fortificant iron; compared to infants of secretor mothers, infants of non-secretor mothers may be more vulnerable to the adverse effect of iron but also benefit more from the co-provision of GOS.

## 1. Introduction

Human milk oligosaccharides (HMOs) represent the third most abundant solid milk component after lipids and lactose in human breast milk, with a concentration of 5–10 g/L and more than 200 different structures [1]. The HMO composition of breast milk varies between mothers, differs across geographic regions, and changes over the course of lactation [2,3,4]. HMOs with L-fucose (Fuc) residuals in α-1-2-linkage such as 2-fucosyllactose (2′FL) and lacto-N-fucopentose I (LNFPI) are only present in breast milk of women with a functional α-1-2-fucosyltransferase (FUT2) (the secretor phenotype), but are absent in breast milk of women with a homozygous mutation in the FUT2 gene (the non-secretor phenotype) [5]. Frequency of the secretor phenotype is estimated at ~80% worldwide but varies between geographical regions [3,6]. The secretor phenotype predicts a higher risk for viral infections (e.g., norovirus [7,8], rotavirus [9,10,11] and respiratory viruses [12]), while the non-secretor phenotype predicts risk for enterotoxigenic *Escherichia coli* (ETEC) infection [13]. Two recent large cohort studies in the United Kingdom and Canada found no association between maternal secretor status and the overall maternal gut microbiota composition [14,15].

Most HMOs are not absorbed in the gastrointestinal tract of the breastfed infant and reach the colon intact, where they can act as prebiotics [16]. *Bifidobacterium* spp., with strain specific ability, and *Bacteroides* spp., both express enzymes for efficient use of HMOs as a carbon source [16]. The effects of HMOs on the breastfed infant gut microbiota likely depend on the breast milk HMO profile, and because there are differences in HMO composition between secretors and non-secretors [4,17], the effects may vary by maternal secretor status [18,19,20]. Abundances of *Bifidobacterium* were found to be higher among infants of secretor mothers [18,19,20], but not all studies agree [21,22,23]. Moreover, HMOs may act as anti-adhesive antimicrobials by operating as receptors for potential pathogenic bacteria (e.g., pathogenic *E. coli*, *Campylobacter jejuni* and *Helicobacter pylori*) and viruses [1,24,25,26,27,28]. High relative abundances of α-1-2-linked-fucosyloligosaccharides and a higher ratio of α-1-2-linked- to non-α-1-2-linked-fucosyloligosaccharides predict lower risk of infant diarrhea [29,30]. Recent studies suggest HMO composition might affect infant growth [31,32,33], but not all studies agree [34].

To treat or prevent iron deficiency anemia (IDA) many infants in low-income countries receive iron fortificants between 6–24 months of age [35]. However, iron is an essential micronutrient for most gut bacteria and is important for virulence and colonization of potential enteropathogens [36]. Two trials in Kenyan infants have reported lower abundances of Bifidobacteria and higher abundances of enteropathogens and enteropathogenic *E. coli* in infants receiving iron-containing micronutrient powders (MNPs) [37,38]. We have recently shown that co-provision of prebiotic galacto-oligosaccharides (GOS) in iron-containing MNPs mitigates most of the adverse effects of the iron on the infant gut microbiota and increases iron absorption [37,39]. As ‘natural prebiotics’, HMOs could provide similar protection from the adverse effects of iron fortificants on the infant gut microbiota, and these protective effects could depend on specific HMO composition of breast milk and maternal secretor status.

Therefore, our study aim was to: (1) determine breast milk HMO concentrations and secretor status of lactating Kenyan mothers and investigate the effect of maternal secretor status on the maternal and infant gut microbiota composition and gut inflammation, as well as on infant iron status and growth; and (2) investigate the effect of maternal secretor status on the infant response to iron fortificants with or without co-provision of GOS, in terms of effects on the infant gut microbiota, enteropathogen abundances, inflammation, iron status, growth and infectious morbidity. We hypothesized that: (1) maternal secretor status would not affect the maternal gut microbiota but would affect the infant gut microbiota, with infants of secretor mothers having higher abundances of *Bifidobacterium* and *Bacteroides* but lower abundances of enteropathogens; and (2) the adverse effect of iron on the infant gut microbiota, as well as the beneficial effects of co-provision of GOS on the infant gut microbiota and on iron absorption, would be stronger among infants of non-secretor mothers.

## 2. Materials and Methods

### 2.1. Study Design

This study was nested within a 4-month, double-masked randomized controlled intervention trial, conducted between October 2014 and January 2016 in southern coastal Kenya; its methods have been previously described in detail [37]. The intervention trial was approved by the ethics and research committees of the Kenyatta National Hospital/University of Nairobi, Kenya (P521/10/2013) and the Zurich Cantonal Ethical Commission (2014–0232); this sub-study was approved by the Kenyatta National Hospital/University of Nairobi, Kenya (P521/10/2013). The participating mothers gave informed consent for themselves and their infant by either a written signature or a fingerprint. An independent Data Safety Monitoring Board monitored the intervention study. The study was registered at ClinicalTrials.gov; identifier: NCT02118402.

In the intervention trial, we included generally healthy infants 6.5–9.5 months of age who had received no vitamin and mineral supplements 8 weeks and no antibiotics 10 weeks prior to study entry, who had a hemoglobin (Hb) >70 g/L, and Z-scores weight-for-age (WAZ) and weight-for-length (WLZ) both >−3. Enrolled infants (*n* = 155) were randomly assigned to three intervention groups using a computer-generated list and three color codes: (1) the control group, receiving daily for 4 months a MNP containing several minerals and vitamins but no iron and 10.5 g of maltodextrin as a carrier in the sachet, (2) the Fe group, receiving daily for 4 months the same MNP but with 2.5 mg iron as sodium iron ethylenediaminetetraacetate (NaFeEDTA) and 2.5 mg iron as ferrous fumarate, and (3) the FeGOS group, receiving daily for 4 months the alike MNP as the Fe group, except the maltodextrin was replaced with 10.5 g of 75% GOS (Vivinal GOS 75 Powder, Friesland Campina, Wageningen, The Netherlands). Table 1 shows the composition of the MNPs used in the study. Study participants and investigators were masked to group assignment. Weekly for 4 months we dispensed seven MNP sachets and 2 kg of unfortified, refined maize flour to the mothers. During the weekly visits, we assessed compliance by questioning the caregiver and collecting the previous week’s used and unused MNP sachets, and infant morbidity over the previous seven days using a forced-choice questionnaire on days affected by: diarrhea (defined as ≥ 3 loose stools in a day) and/or mucus in stool; respiratory tract infections (RTIs) (defined as cough and/or difficult or rapid breathing); fever; and/or other illness.

All of the mothers of the participating infants (*n* = 155) were asked to join this sub-study; 80 mothers gave informed consent and were included in this sub-study. Fecal samples from all included mothers and infants were collected at baseline, after 3 weeks and after 4 months of the intervention for determination of the gut microbiota by 16S rDNA sequencing, enteropathogens by quantitative polymerase chain reaction (qPCR) and fecal calprotectin. Mothers were carefully instructed on fecal sample collection and were given plastic diapers (for collection of the infant fecal sample), spatulas and screw-cap plastic containers containing a carbon dioxide generator system to create an anaerobic atmosphere (Microbiology Anaerocult A mini, Merck, Darmstadt, Germany). The fecal samples were collected at home into the containers. The study team aliquoted and stored the fecal sample at −20 °C the same day until further analysis. Breast milk samples for analysis of HMOs were obtained by manual expression by the mother into a clean plastic container. The samples were kept cool until the study team aliquoted and stored them at −20 °C the same day until further analysis. From all mothers (*n* = 80) we collected a breast milk sample at one time point; the time point of this sample collection was not standardized. From a sub-group of all mothers (*n* = 16) we collected breast milk samples at two different time points of lactation for determination of potential changes in HMO composition over time; the time points of collection of these two samples were standardized in that the second sample was collected 3 months later in lactation than the first sample. A blood sample from all infants was collected at baseline and after 4 months of the intervention for determination of Hb, plasma ferritin (PF), soluble transferrin receptor (sTfR), C reactive protein (CRP), alpha-glycoprotein (AGP) and intestinal fatty acid binding protein (I-FABP). At baseline and after 4 months of the intervention we measured infant weight and length. At baseline we recorded demographic characteristics, brief medical history and feeding habits of the infant and mothers, including information on breastfeeding, using a questionnaire and local health records.

### 2.2. Laboratory Methods

Breast milk samples: HMO composition in maternal breast milk samples was quantitatively determined using high-performance anion-exchange chromatography coupled with pulse amperometric detection (HPAE-PAD) [40,41]; details are given in the Supplementary Material (Supplementary Methods and Table S1).

Fecal samples: Details on the protocol used for DNA extraction of infant and maternal fecal samples are given in the Supplementary Material (Supplementary Methods). Using a 2-step PCR, barcoded amplicons from the V3–V4 region of 16S rRNA genes were generated. For initial amplification of the V3–V4 part of the 16S rRNA we used universal primers appended with Illumina adaptor sequences. PCR products were purified, checked on a Bioanalyzer (Agilent) and quantified. Illumina MiSeq with the paired-end (2×) 300 bp protocol and indexing was used for sequencing, followed by de-multiplexing and quality control. Details of the method are given in the Supplementary Material (Supplementary Methods). Selected enteropathogenic bacteria (*Clostridium difficile*, *Clostridium perfringens,* enterohemorrhagic *E. coli* with shiga toxin 1 (EHEC *stx1*), enterohemorrhagic *E. coli* with shiga toxin 2 (EHEC *stx2*), enteropathogenic *E. coli* with the attaching and effacing gene (EPEC *eaeA*), enterotoxigenic *E. coli* with heat-stable enterotoxin (ETEC ST), enterotoxigenic *E. coli* with heat-labile enterotoxin (ETEC LT), *Salmonella* spp. and *Staphylococcus aureus*) were targeted in infant and maternal fecal DNA using qPCR. Details of the qPCR method are given in the Supplementary Material (Supplementary Methods). Primers used for qPCR [42,43,44] are given in Table S2. We measured fecal calprotectin in all infant fecal samples using the Calprest ELISA assay for fecal samples (Eurospital, Trieste, Italy).

Blood samples: We collected a venous blood sample (3 mL) from the infant at baseline and 4 months. Using a HemoCue 300 analyzer (HemoCue, Angelholm, Sweden) we measured Hb on the day of collection. After centrifugation to separate plasma, we froze the plasma on the day of collection until further analysis of PF, sTfR, CRP and AGP using a multiplex immunoassay [45]. We measured plasma I-FABP using a commercially available ELISA (Hycult Biotech, Uden, The Netherlands).

### 2.3. Data and Statistical Analysis

We defined total fucosylated HMOs as the sum of 2′FL, 3-fucosyllactose (3′FL), LNFPI, lacto-N-fucopentaose II (LNFPII) and lacto-N-fucopentaose III (LNFPIII); total sialylated HMOs as the sum of 6-sialyllactose (6′SL), 3-sialyllactose (3′SL), sialyllacto-N-tetraose d (LSTd), sialyllacto-N-tetraose a (LSTa) and disialyl-lacto-N-teraose (DSLNT); and total non-fucosylated and non-sialylated HMOs as the sum of lacto-N-neotetraose (LNnT), lacto-N-teraose (LNT) and lacto-N-neohexaose (LNnH). We analyzed absolute and relative concentration of HMOs; details are given in the Supplementary Material (Supplementary Methods). We defined a mother as being non-secretor if there were no detectable concentrations of α-1-2-linked fucosylated HMOs (2′FL and LNFPI) in her breast milk sample. Z-scores for length-for-age (LAZ), WAZ and WLZ were calculated using the WHO Anthro software (Version 3.2.2.1). We calculated body iron stores (BIS) from the sTfR/PF ratio using the following equation: BIS (mg/kg) = ((log(sTfR / PF) − 2.8229) / 0.1207 [46]. We calculated total BIS by multiplying BIS with infant weight.

We analyzed 16S rRNA gene sequences using a workflow based on Qiime 1.8 [47]. We performed operational taxonomic unit (OTU) clustering (open reference), taxonomic assignment and reference alignment with the pick_open_reference_otus.py workflow script of Qiime, using uclust as clustering method (97% identity) and GreenGenes v13.8 as reference database for taxonomic assignment. Reference-based chimera removal was done with Uchime [48]. The RDP classifier version 2.2 was performed for taxonomic classification [49]. We performed statistical tests as implemented in SciPy (https://www.scipy.org/), downstream of the Qiime-based workflow. We used the Fluidigm Real-Time PCR Analysis Software and Excel (Microsoft Office 2010) for processing the qPCR data, including melting curve analysis. Details of the methods are given in the Supplementary Material (Supplementary Methods).

We performed multivariate redundancy analyses (RDAs) on the infant and maternal gut microbiota composition as assessed by 16S rRNA gene sequencing in Canoco version 5.11 using default settings of the analysis type “Constrained” [50]; details of the method and comparisons are given in the Supplementary Material (Supplementary Methods). We tested for between group differences at baseline (secretor vs. non-secretor mothers and infants of secretor mothers vs. infants of non-secretor mothers, respectively) in alpha diversity and abundance of the taxa of primary interest (Bacteroidetes, *Bifidobacterium*, *Clostridiales*, *Enterobacteriaceae* and *Lactobacillus*) and of all taxa, using Wilcoxon rank-sum tests with FDR correction for multiple testing. We tested for differences in alpha diversity (PD whole tree, and for *Bifidobacterium* diversity analysis also Shannon index), phylogenetic distance (weighted UniFrac within individuals between baseline and 3 weeks sample, and between baseline and 4 months) and abundance of the taxa of primary interest and of all taxa, at baseline, 3 weeks and 4 months between the intervention–secretor-status groups (control group infants of secretor mothers (control-S) and of non-secretor mothers (control-NS), Fe group infants of secretor mothers (Fe-S) and of non-secretor mothers (Fe-NS), FeGOS group infants of secretor mothers (FeGOS-S) and of non-secretor mothers (FeGOS-NS)), using Kruskal–Wallis tests with Dunn’s post hoc test to adjust for multiple comparisons. Details of the comparisons are given in the Supplementary Material (Supplementary Methods).

We analyzed demographic, anthropometric and biochemical data using the R statistical programming environment (R. 3.4.1 software; R Core Team). Normally distributed data are shown as mean ± SD and non-normally distributed data as median (IQR). We tested for changes over time of lactation in HMO concentration using Wilcoxon signed-rank tests. We tested for between group differences (secretor vs. non-secretor mothers and infants of secretor mothers vs. infants of non-secretor mothers, respectively) of biochemical data at baseline using *t*-tests or Wilcoxon rank-sum tests. The interaction between maternal secretor status (secretor and non-secretor) and intervention groups (control, Fe and FeGOS) was investigated by fitting linear mixed-effect models using CRAN package lme. We defined the fixed effects on the variance as time, secretor status, intervention group, time-by-secretor-status, time-by-intervention-group, secretor-status-by-intervention group and time-by-secretor-status-by-intervention-group, and the random structure as the subject to control between-subject differences. Post hoc analyses were performed to investigate the effect of time within group (intervention–secretor-status groups). Details of the methods and comparisons are given in the Supplementary Material (Supplementary Methods). We evaluated infant morbidity using longitudinal prevalence ratio (LPR) [51,52]; details of the method are given in the Supplementary Material (Supplementary Methods).

In all analyses, *p* values < 0.05 were considered statistically significant.

## 3. Results

### 3.1. Characteristics of the Study Population

Eighty mother–infant pairs were enrolled; 5 mother–infant pairs were excluded due to inadequate fecal and/or breast milk sample collection. Data from 75 mother–infant pairs were included in the data analyses. Characteristics of the study population at baseline, overall and by maternal secretor status are given in Table 2. Median duration of exclusive breastfeeding was 6 months. At baseline, all infants were already receiving complementary foods, mainly maize porridge, and all were still partially breastfed. During the period of the intervention study, more than 50% of the mothers reported breastfeeding >5 times per day and the remaining mothers reported breastfeeding between 1–5 times per day. During the intervention study, compliance with the MNP sachets was 96%, 97% and 95% in the control, Fe and FeGOS groups, respectively.

### 3.2. Maternal Secretor Status, HMO Composition and the Maternal Gut Microbiota

**Figure 1:**
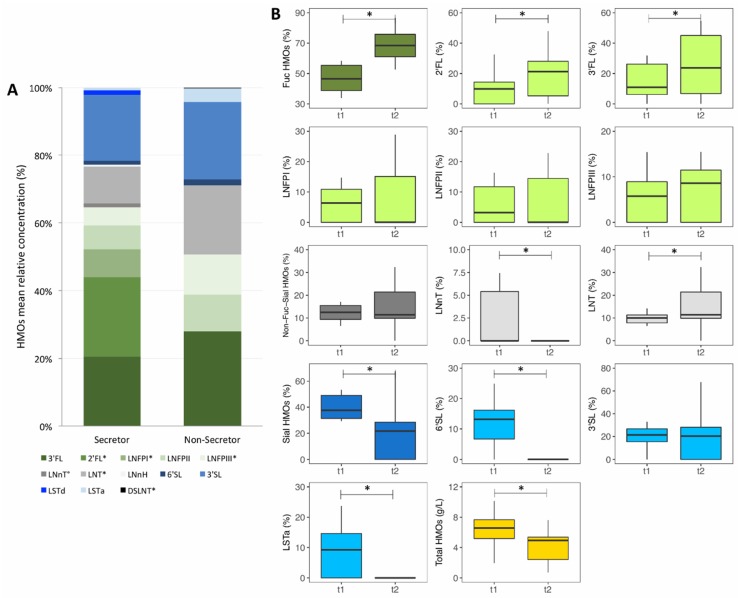
Concentration of human milk oligosaccharides (HMOs) in breast milk samples of Kenyan mothers. (**A**) Mean relative concentration of HMOs in breast milk samples of Kenyan mothers (*n* = 75), by secretor status. (**B**) Absolute concentration of total HMOs (g/L) and relative concentration (%) of single HMOs, Sial HMOs (total sialylated HMOs), Fuc HMOs (total fucosylated HMOs), Non-Fuc-Sial HMOs (total non-fucosylated and non-sialylated HMOs) in breast milk samples of Kenyan mothers (*n* = 16) over 3 months of lactation. 2′FL, 2-fucosyllactose; 3′FL, 3-fucosyllactose; LNFPI, lacto-N-fucopentaose I; LNFPII, lacto-N-fucopentaose II; LNFPIII, lacto-N-fucopentaose III; LNnT, lacto-N-neotetraose; LNT, lacto-N-teraose; LNnH, lacto-N-neohexaose; 6′SL, 6-sialyllactose; 3′SL, 3-sialyllactose; LSTd, sialyllacto-N-tetraose d; LSTa, sialyllacto-N-tetraose a; and DSLNT, disialyl-lacto-N-teraose. Differences between groups and time points were tested using Wilcoxon rank-sum tests and Wilcoxon signed-rank tests, respectively; * *p* < 0.05, ° *p* < 0.10.

Maternal secretor status and HMO composition: A breast milk sample was collected from 75 mothers at one time point of lactation. Median (IQR) lactation stage (month postpartum) at the time of this breast milk sample collection was for all mothers 14.1 (11.2–16.5), for secretor mothers 13.8 (11.2–15.9) and for non-secretor mothers 16.1 (11.4–18.0). Fifty-four of the mothers (72%) were identified as being secretors and 21 (28%) as non-secretors. HMO concentrations among all mothers and by secretor status are given in Table S3. Mean relative HMO concentrations by secretor status are shown in Figure 1A. Only breast milk of secretor mothers contained 2′FL and LNFPI, while there was no measurable concentration of these two HMOs in breast milk of non-secretor mothers. Absolute and relative concentrations of LNFPIII and relative concentration of LNT were higher among non-secretor mothers compared to secretor mothers (*p* = 0.015 and *p* = 0.035, respectively). The higher absolute concentrations of LNFPII and LNT among non-secretor mothers compared to secretor mothers failed to reach significance (*p* = 0.097 and *p* = 0.075, respectively).

HMO abundances over the course of lactation: From a sub-group of all mothers (*n* = 16) we collected breast milk samples at two different time points of lactation. Median (IQR) lactation stage (months postpartum) when the first and second breast milk samples were collected was 7.9 (7.8–8.5) and 10.9 (10.7–11.5). Absolute and relative HMO concentrations at the two time points are given in Table S4 and are shown in Figure 1B. Absolute concentrations of LNnT, 6′SL, LSTa, total sialylated HMOs and total HMOs decreased over lactation (*p* = 0.022, *p* = 0.003, *p* = 0.006, *p* = 0.001 and *p* = 0.034, respectively). Absolute concentration of non-fucosylated and non-sialylated HMOs decreased among secretor mothers (*p* = 0.042). Relative concentrations of LNnT, 6′SL, LSTa and of total sialylated HMOs decreased over lactation (*p* = 0.022, *p* = 0.003, *p* = 0.006 and *p* = 0.001, respectively). Relative concentration of 3′SL decreased among secretor mothers (*p* = 0.042). In contrast, relative concentrations of 2′FL, 3′FL, LNT and of total fucosylated HMOs increased over lactation (*p* = 0.003, *p* = 0.004, *p* = 0.025 and *p* = 0.002, respectively).

Maternal gut microbiota composition by secretor status, by 16S rDNA sequencing: Per sample on average 16657 bacterial 16S rDNA sequences were analyzed by 16S rDNA sequencing. At baseline, among all mothers, among secretor mothers and among non-secretor mothers, respectively the microbiota was composed of the phylum Actinobacteria (8.9%, 9.0% and 8.7% of the 16S rDNA reads), Firmicutes (73.7%, 73.3% and 74.6%), Bacteroidetes (12.6%, 12.5% and 12.8%) and Proteobacteria (3.6%, 4.1% and 2.5%). Details on the microbiota composition are shown in Figure S1A–C. There were no significant differences in the RDA on all OTUs and in the RDA on *Bifidobacterium* OTUs, in phylogenetic diversity (alpha-diversity; PD whole tree), or in any individual taxa at baseline between secretor mothers and non-secretor mothers.

Maternal gut microbiota by secretor status, by qPCR: abundances and occurrence of gut pathogens: From all maternal fecal samples analyzed (*n* = 66), we detected *C. perfringens* in 67% (median log gene copies/g feces in samples with detected abundances: 4.4 (IQR: 4.0–5.1)), EPEC *eaeA* in 39% (5.1 (4.4–5.5)), ETEC ST in 23% (4.1 (3.9–4.5)), EHEC *stx2* in 18% (4.5 (4.1–5.4)), ETEC LT in 14% (5.7 (5.0–6.3)), and EHEC *stx1* in 9% (4.1 (3.9–4.3)). *C. difficile* was not detected. Abundance of *C. perfringens* was significantly higher among non-secretor mothers (detected in 70%, 4.9 (4.7–5.6)) compared to secretor mothers (65%, 4.1 (3.8–4.9)) (*p* = 0.028). There was no significant difference in the abundance of the sum of virulence and toxin genes (VTGs) of pathogenic *E. coli* (*eaeA*, LT, ST, *stx1*, *stx2*) between secretor mothers (61%, 5.1 (4.6–5.8)) and non-secretor mothers (50%, 4.4 (4.1–5.6)) (*p* = 0.125) and in the abundance of the sum of all pathogens between secretor mothers (85%, 5.1 (4.3–5.8)) and non-secretor mothers (85%, 5.3 (4.7–5.7)) (*p* = 0.698).

### 3.3. Cross-Sectional Analyses at Baseline: Comparison of Infants of Secretor Mothers and Infants of Non-Secretor Mothers

Infant gut microbiota composition by 16S rDNA sequencing: Per sample on average 24696 bacterial 16S rDNA sequences were analyzed by 16S rDNA sequencing. At baseline, among all infants, among infants of secretor mothers and among infants of non-secretor mothers, respectively the microbiota consisted of the phyla Actinobacteria (68.2%, 66.7% and 72.2% of the 16S rDNA reads, which was mostly represented by the family *Bifidobacteriaceae* (61.8%, 61.1% and 63.6%)), Firmicutes (27.2%, 28.7% and 23.3%; including 10.6%, 10.4% and 11.2% *Clostridiales*, and 3.6%, 4.0% and 2.7% *Lactobacillus*), Bacteroidetes (1.5%, 1.8% and 0.6%) and Proteobacteria (3.0%, 2.7% and 3.7%; with the predominant family *Enterobacteriaceae* (2.9%, 2.6% and 3.7%)). Details are given in Figure S2A–C. There were no significant differences in the RDA on all OTUs and in the RDA on *Bifidobacterium* OTUs, in overall phylogenetic diversity (alpha-diversity; PD whole tree) and *Bifidobacterium* OTUs specific phylogenetic diversity (PD whole tree and Shannon index), or in any individual taxa at baseline between infants of secretor mothers and infants of non-secretor mothers.

Infant gut microbiota by PCR: abundance and occurrence of gut pathogens: From all analyzed infant fecal samples (*n* = 75), we detected *C. perfringens* in 68% (median log gene copies/g feces in samples with detected abundances: 4.6 (IQR: 3.9–5.1)), EPEC *eaeA* in 65% (5.4 (4.8–6.5)), *C. difficile* in 44% (5.6 (4.7–6.0)), ETEC LT in 27% (6.6 (5.8–8.0)), EHEC *stx2* in 19% (4.3 (4.0–4.7)), ETEC ST in 15% (4.4 (4.1–5.8)), *S. aureus* in 13% (4.5 (4.5–5.1)), EHEC *stx1* in 7% (4.3 (3.9–4.7)) and *Salmonella* spp. in 3% (4.5 (4.4–4.6)). Comparing infants of secretor mothers to infants of non-secretor mothers, there were no significant differences in the abundance of the sum of all pathogens (*p* = 0.895), the abundance of the sum of VTGs of pathogenic *E. coli* (*p* = 0.763), or when analyzing all pathogens separately. Secretor status did not predict the sum of all pathogens and VTGs of pathogenic *E. coli* in a linear regression model.

Infant anthropometrics, hematological and inflammation status, plasma I-FABP and fecal calprotectin: There were no significant differences in anthropometrics, Hb, PF, sTfR, CRP, AGP, I-FABP, or fecal calprotectin between infants of secretor mothers and infants of non-secretor mothers (Table 2). In linear regression models, maternal secretor status did not significantly predict infant weight, length, LAZ, WAZ, WLZ, Hb, PF, sTfR, CRP, AGP, I-FABP or fecal calprotectin.

### 3.4. Effect of Maternal Secretor Status on the Infant Response to the Iron and GOS Intervention

**Figure 2:**
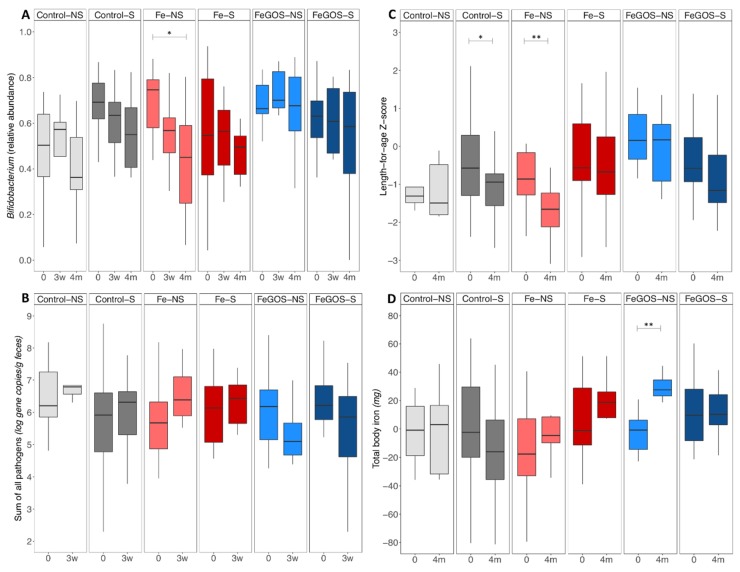
Relative abundances of *Bifidobacterium* and of the sum of virulence and toxin genes of the 10 targeted pathogens, length-for-age Z-score and total body iron, by group. Kenyan infants (*n* = 75) receiving daily a micronutrient powder containing either no iron (Control–) (*n* = 26), containing 5 mg of iron (Fe–) (*n* = 24), or containing 5 mg of iron and 7.5 g of galacto-oligosaccharides (FeGOS–) (*n* = 25), and being breastfed by either a non-secretor mother (–NS) (*n* = 21) or a secretor mother (–S) (*n* = 54). (**A**) Relative abundance of *Bifidobacterium* by group at baseline (0), 3 weeks (3w) and 4 month (4m) of the intervention. (**B**) Log gene copies/g feces of the sum of all pathogens by group at baseline (0) and 3 weeks (3 w) of the intervention. (**C**) Length-for-age Z-score by group at baseline (0) and 4 month (4m) of the intervention. (**D**) Total body iron (mg) by group at baseline (0) and 4 month (4m) of the intervention. We assessed an interaction between maternal secretor status and intervention-groups by fitting linear mixed-effect models using CRAN package lme. We defined the fixed effects on the variance as time, secretor-status, intervention-group, time-by-intervention-group, time-by-secretor-status, secretor-status-by-intervention-group, and time-by-secretor-status-by-intervention-group, and the random structure was defined as the subject. There was a significant secretor-status-by-intervention-group effect on *Bifidobacterium* abundance (*p* = 0.021), a significant time-by-secretor-status-by-intervention-group effect (*p* = 0.023) and a significant secretor-status-by-intervention group effect (*p* = 0.022) on length-for-age Z-score, and a significant time-by-intervention-group (*p* = 0.003) and time-by-secretor-status (*p* = 0.016) effect on total body iron. Post hoc analyses were performed to investigate the effect of time within group. Boxes show the median and 25th and 75th percentile; whiskers extend to the furthest data point that is within 1.5 times the IQR. * *p* < 0.05; ** *p* < 0.01.

Infant gut microbiota composition by 16S rDNA sequencing: There were no significant differences in the RDAs on all OTUs and in the RDAs on *Bifidobacterium* OTUs, in phylogenetic diversity (alpha-diversity; PD whole tree), phylogenetic diversity on *Bifidobacterium* OTUs (PD whole tree), and Shannon Index on *Bifidobacterium* OTUs, or in beta-diversity (weighted Unifrac) at baseline, 3 weeks and 4 months when comparing the intervention–secretor-status groups (control-S (*n* = 21), control-NS (*n* = 5), Fe-S (*n* = 16), Fe-NS (*n* = 8), FeGOS-S (*n* = 17) and FeGOS-NS (*n* = 8)). In the mixed-effect model on *Bifidobacterium* abundances there was a significant secretor-status-by-intervention-group interaction (*p* = 0.021). Post hoc tests for within intervention–secretor-status group differences showed a significant decrease in *Bifidobacterium* abundances from baseline to 4 months in the Fe-NS group (*p* = 0.028). Relative abundances of *Bifidobacterium* at baseline, 3 weeks and 4 months, by intervention–secretor-status groups are shown in Figure 2A. In the cross-sectional analyses at baseline, 3 weeks and 4 months and in the longitudinal analyses from baseline to 3 weeks and from baseline to 4 months there were no significant differences in any other taxa when comparing the intervention–secretor-status groups.

Infant gut microbiota by qPCR: abundances and occurrence of gut pathogens: There was a significant time-by-intervention-group (*p* = 0.007) and a secretor-status-by-intervention-group interaction (*p* = 0.096) on the sum of all pathogens from baseline to 3 weeks. Log gene copies/g feces of the sum of all pathogens at baseline and 3 weeks, by intervention–secretor-status groups are shown in Figure 2B. Within the Fe intervention group infants of non-secretor mothers (Fe-NS) have a greater increase in the sum of all pathogens. Within the FeGOS intervention groups the decrease in the sum of pathogens was particularly pronounced among infants of non-secretor mothers (FeGOS-NS). Post hoc tests for within group differences were not statistically significant after adjusting for multiple testing.

Fecal calprotectin and plasma I-FABP: There was a time-by-secretor-status-by-intervention-group (*p* = 0.086), time-by-secretor-status (*p* = 0.066) and time-by-intervention-group (*p* = 0.068) effect on fecal calprotectin from baseline to 3 weeks and a time-by-secretor-status effect from baseline to 4 months (*p* = 0.017). Post hoc tests on within group differences from baseline to 3 weeks and from baseline to 4 months showed a significant decrease in the Control-NS group (*p* = 0.008 and *p* = 0.014, respectively). There was no significant time-by-secretor-status-by-intervention-group, time-by-secretor-status or secretor-status-by-intervention-group effect on I-FABP.

Infant anthropometrics: There was a significant time-by-secretor-status-by-intervention-group effect (*p* = 0.023) and secretor-status-by-intervention-group effect (*p* = 0.022) on LAZ and a significant secretor-status-by-intervention-group effect (*p* = 0.018) on WAZ. Post hoc tests on within group differences from baseline to 4 months showed a significant decrease in LAZ in the Control-S and Fe-NS groups (*p* = 0.031 and *p* = 0.002, respectively). LAZ at baseline and 4 months of the intervention, by intervention–secretor-status groups is shown in Figure 2C.

Hemoglobin and iron status: There was a significant time-by-intervention-group effect on Hb (*p* = 0.036) but no significant time-by-secretor-status-by-intervention-group, time-by-secretor-status or secretor-status-by-intervention-group effect. The time-by-secretor-status effect on PF was borderline significant (*p* = 0.052) and there was a significant time-by-intervention-group and secretor-status-by-intervention-group effect on sTfR (*p* = 0.002 and *p* = 0.041, respectively). There was a significant time-by-intervention-group effect and time-by-secretor-status effect on BIS (*p* = 0.011 and *p* = 0.023, respectively) and on total BIS (*p* = 0.003 and *p* = 0.016, respectively). Post hoc tests on within group differences from baseline to 4 months showed a significant increase in PF, BIS and total BIS in the FeGOS-NS group (*p* = 0.043, *p* = 0.012 and *p* = 0.002, respectively). Total BIS at baseline and 4 months, by intervention–secretor-status groups is shown in Figure 2D.

Infant morbidity: Longitudinal prevalence (percentage of weeks with illness) of diarrhea and/or mucus in the stool was higher in the Control-NS group (23.8%) compared to the Control-S group (10.4%) (LPR = 2.28, 95% CI 1.38 to 3.77, *p* = 0.001) but was not different between FeGOS-NS (16.4%) and FeGOS-S (11.0%) (*p* = 0.132) and between Fe-NS (9.4%) and Fe-S (12.5%) (*p* = 0.376). Longitudinal prevalence of RTIs was higher in Fe-S (19.5%) compared to Fe-NS (10.9%) (LPR = 0.56, 95% CI 0.32 to 0.97, *p* = 0.040) but was not different between Control-NS (10.0%) and Control-S (14.0%) (*p* = 0.359) and between FeGOS-NS (18.1%) and FeGOS-S (12.5%) (*p* = 0.135).

## 4. Discussion

Our main findings are: (1) a secretor prevalence of 72%; (2) significant differences in concentration of HMOs between secretor and non-secretor mothers and over time of lactation; (3) no significant differences in the overall gut microbiota composition, phylogenetic diversity, abundances of taxa of primary interest and abundances of enteropathogens comparing secretor to non-secretor mothers and infants of secretor to infants of non-secretor mothers, respectively, with the exception of a higher abundance of *C. perfringens* among non-secretor compared to secretor mothers; (4) a significant secretor-status-by-intervention-group interaction on *Bifidobacterium* abundance, LAZ and WAZ, and sTfR; and (5) among infants in the intervention control group, a higher longitudinal prevalence of diarrhea among infants of non-secretor compared to infants of secretor mothers.

Secretor prevalence (72%) in our study is at the lower end of published prevalence data. Worldwide secretor frequency is estimated at ~80%, but varies across different geographic areas [3,6]: 67–95% in the United States [3,7,18,53,54,55], 64–87% in the United Kingdom, Spain, Finland, Sweden and Italy [2,14,17,56,57,58,59,60], 85% in India [56], 77% and 80% in China [20,61], and 51–81% in Africa (Burkina Faso, Ethiopia, Ghana, Kenya, South Africa, The Gambia and Malawi) [3,54,60,62,63]. Secretor prevalence in urban and rural Gambia is 85% and 65%, respectively, and in urban and rural Ethiopia is 78% and 65%, respectively [3]. In urban Kenya (Nakuru), secretor prevalence is 81% [3], and like the above data from The Gambia and Ethiopia, our secretor prevalence in rural Kenya is lower, at 72%.

Our findings agree with previous studies reporting higher concentration of total HMOs [2,17,64] and higher abundances of fucosylated HMOs [2,54,64] in secretor compared to non-secretor mothers, but not all studies agree [18,54]. Two studies reported higher abundances of sialylated HMOs in non-secretor compared to secretor mothers [2,54], while others (as in our study) found no difference [64]. Higher abundances of 2′FL, LNFPI and 3′FL have been reported in secretor compared to non-secretor mothers [2,17], but others found 3′FL to be higher in non-secretor mothers [4]. Our findings agree with previous studies reporting higher amounts of LNT, LNFPII and LNFPIII in non-secretor mothers [2,4,17]. Our findings also agree with previous studies reporting total HMO concentrations decrease over the course of lactation [54]. The relatively low median total HMO concentration of 4.5 g/L in our study, compared to reported values of 5–10 g/L [1], is likely explained by our sampling in the late stage of lactation. Our finding of decreases over lactation of LNnT, 6′SL, LSTa and total sialylated HMOs but increases of 3′FL, 2′FL, LNT and total fucosylated HMOs are in general agreement with previous data, although findings vary [2,4,20,54,65].

To our knowledge, our study is the first to investigate the effect of maternal secretor status among mothers and infants living in rural Kenya, an area with low hygiene conditions and a high burden of infectious diseases. Our findings show that maternal secretor status does not have a major impact on the gut microbiota of the mothers. Previous studies have linked maternal secretor status to a higher risk for viral infections, including norovirus [7,8,66], rotavirus [9,10,11,58,67,68] and respiratory viruses [12] in women. In contrast, maternal non-secretor status has been linked with symptomatic ETEC infection in women [13]. In our study, there were no differences in abundance of enteropathogens comparing secretor to non-secretor mothers, with the exception of higher *C. perfringens* among non-secretor mothers. Our findings agree with two cohort studies in the United Kingdom (*n* = 1503) and Canada (*n* = 1190), reporting no association between maternal secretor status and the overall gut microbiota composition, microbiota diversity and relative abundance of taxa in women [14,15], while others reported differences in the gut microbiota comparing secretor and non-secretor women [53,56,58].

Our findings also show that secretor status does not have a major impact on the infant gut microbiota in this setting. Some studies have found differences in the gut microbiota of infants of secretor mothers compared to infants of non-secretor mothers [18,19,20], but others have not [21,22,23]. Our findings agree with studies from Finland, India and the United States reporting no differences in the infant gut microbiome based on maternal secretor status [21,22,23] and with studies showing no association between infant microbiota alpha-diversity and maternal secretor status [19,20]. Studies in Australian toddlers, and from younger infants in China and the United States, reported higher abundance of *Bifidobacterium* among children of secretor mothers [18,19,20]; this was not confirmed in our study.

In our study, during the 4 months of intervention, among infants in the control group, longitudinal prevalence of diarrhea and/or mucus in the stool was higher among infants of non-secretor mothers compared to infants of secretor mothers. Two previous studies found lower incidence of diarrhea among infants breastfed with milk being high in relative abundance of α-1-2-linked-fucosyloligosaccharides or having a high ratio of α-1-2-linked- to non-α-1-2-linked-fucosyloligosaccharides [29,30]. Higher relative amounts of LNT and lower levels of LNFPI and LNFPII were found in milk of Gambian mothers whose infant was sick compared to milk of mothers whose infant was not sick [32].

There were no significant differences in anthropometrics at baseline between infants of secretor mothers and infants of non-secretor mothers in our study. Recent studies suggest breast milk HMO composition may affect infant growth [31,32,33], but not all studies agree [34]. Breast milk from non-secretor mothers from severely stunted infants in Malawi showed different HMO concentrations compared to breast milk from non-secretor mothers from healthy infants, with lower concentrations of sialylated and fucosylated HMOs [31].

Importantly, the differing breast milk HMO profile of secretor and non-secretor mothers appeared to modulate the impact of the iron and GOS intervention. Infants in the Fe group and of non-secretor mothers were particularly vulnerable to the decrease in abundance of *Bifidobacterium* and to the increase in the sum of all pathogens. However, infants of non-secretor mothers benefited the most from the co-provision of GOS (FeGOS-NS group) in maintaining high abundances of *Bifidobacterium* and reducing enteropathogens. Moreover, there was a secretor-status-by-intervention-group interaction on iron status: within the FeGOS intervention group, there were greater improvements in PF, sTfR and BIS among infants of non-secretor mothers, suggesting the enhancing effect of GOS on iron absorption [39] might be stronger in infants of non-secretor mothers.

Our study has several strengths: (1) the quantitative analysis of HMOs in breast milk samples including investigation of changes over duration of lactation; (2) the extensive characterization of the infant and maternal gut microbiota using both 16S rDNA sequencing and qPCR for selected enteropathogens; and (3) inclusion of mothers and infants from an area of poor hygiene with a high burden of diarrhea and other infectious diseases. Our study was limited in that we did not standardize the time of the day when the breast milk samples were collected nor on whether to collect fore or hind milk, we did not investigate individual *Bifidobacterium* spp., and we enrolled a relatively small sample size.

## 5. Conclusions

In conclusion, maternal secretor status modulated the effects of the iron and GOS intervention: compared to infants of secretor mothers, infants of non-secretor mothers may be more vulnerable to the adverse effect of fortificant iron on the gut microbiota, resulting in decreased abundances of *Bifidobacterium* and increased abundances of enteropathogens, but also benefit more from the co-provision of GOS in terms of beneficial effects on the gut microbiota and improving iron status. Future studies would be valuable to investigate the effect of specific HMOs on iron absorption and their effects on the gut microbiota when given with fortificant iron.

## Figures and Tables

**Table 1:** Composition of the micronutrient powder formulations used in this study.

Components	Control Group	Fe Group	FeGOS Group
	Amount per sachet	Amount per sachet	Amount per sachet
Vitamin A	400 µg	400 µg	400 µg
Vitamin D	5 µg	5 µg	5 µg
Tocopherol Equivalents	5 mg	5 mg	5 mg
Thiamine	0.5 mg	0.5 mg	0.5 mg
Riboflavin	0.5 mg	0.5 mg	0.5 mg
Vitamin B_6_	0.5 mg	0.5 mg	0.5 mg
Folic Acid	90 µg	90 µg	90 µg
Niacin	6 mg	6 mg	6 mg
Vitamin B_12_	0.9 µg	0.9 µg	0.9 µg
Vitamin C	30 mg	30 mg	30 mg
Copper	0.56 mg	0.56 mg	0.56 mg
Iodine	90 µg	90 µg	90 µg
Selenium	17 µg	17 µg	17 µg
Zinc	4.1 mg	4.1 mg	4.1 mg
Phytase	190 FTU	190 FTU	190 FTU
Maltodextrin	10.5 g	10.5 g	
Galacto-oligosaccharides			10.5 g
Iron (as ferrous fumarate)		2.5 mg	2.5 mg
Iron (as NaFeEDTA)		2.5 mg	2.5 mg

FTU, phytase unit; NaFeEDTA, sodium iron ethylenediaminetetraacetate; GOS, galacto-oligosaccharides.

**Table 2:** Characteristics of the study population at baseline. Age, parity, gender, anthropometrics, hematological and inflammation status, fecal calprotectin and plasma I-FABP. Differences in these variables among Kenyan mothers (*n* = 75) and Kenyan infants (*n* = 75) at baseline, by maternal secretor status.

	All (*n* = 75)	Secretor (*n* = 54)	Non-Secretor (*n* = 21)
**Mothers**			
Age (y) ^1^	26 (22–30) ^3^	25 (20–29)	28 (25–33)
Parity (n) ^2^	3 (1,10) ^4^	3 (1,10)	4 (1,9)
**Infants**			
Age (mo)	7.2 (7.0–8.2)	7.2 (7.0–8.2)	7.1 (7.0–7.9)
Gender (m/f) (*n* (%))	34 (45%)/41 (55%)	25 (47%)/29 (54%)	9 (43%)/12 (57%)
Weight (kg)	7.5 (7.0–8.5)	7.5 (7.0–8.5)	7.5 (7.0–8.2)
Length (cm)	67.5 (66.0–69.5)	67.5 (66.0–69.4)	68.0 (66.0–69.5)
WAZ	−0.43 ± 1.15 ^5^	−0.35 ± 1.18	−0.63 ± 1.06
WLZ	−0.18 ± 1.25	−0.07 ± 1.26	−0.46 ± 1.22
LAZ	−0.40 ± 1.09	−0.40 ± 1.09	−0.39 ± 1.12
Hemoglobin (g/L)	104 (97–111)	104 (97–113)	104 (98–107)
Plasma ferritin (μg/L)	16.5 (10.0–31.6)	17.2 (10.3–38.4)	15.8 (9.7–24.6)
Soluble transferrin receptor (mg/L)	11.0 (8.6–14.8)	10.2 (8.3–13.8)	12.6 (10.1–16.8)
C reactive protein (mg/L)	1.1 (0.5–6.0)	1.1 (0.4–5.7)	1.1 (0.6–10.8)
Alpha-glycoprotein (g/L)	1.0 (0.7–1.7)	1.0 (0.7–1.8)	0.9 (0.6–1.7)
Fecal calprotectin (μg/g)	228.9 (132.7–347.5)	218.3 (129.9–324.4)	281.1 (136.5–409.4)
I-FABP (pg/mL)	822.6 (677.3–1327.9)	861.0 (688.8–1420.0)	753.2 (527.2–1203.7)

^1^ All, *n* = 74, age missing for one mother in the secretor group. ^2^ Number of infants including infant enrolled into this study. ^3^ Median (IQR), all such values. ^4^ Median (min, max), all such values. ^5^ Mean ± SD, all such values. WAZ, weight-for-age Z-score; WLZ, weight-for-length Z-score; LAZ, length-for-age Z-score; I-FABP, intestinal fatty acid binding protein. Between group differences (secretor vs. non-secretor) were tested using t-tests or Wilcoxon rank-sum tests. There were no significant between group differences.

## Supplementary Materials

The following are available online at https://www.mdpi.com/2072-6643/11/11/2596/s1, supplementary material: Supplementary Methods and Supplementary Results including Table S1: Gradient elution program, Table S2: Primers used for quantitative polymerase chain reaction, Table S3: Absolute and relative concentration of human milk oligosaccharides (HMOs) in breast milk samples of Kenyan mothers (*n* = 75), all and by secretor status, Table S4: Absolute and relative concentration of human milk oligosaccharides (HMOs) in breast milk samples of Kenyan mothers (*n* = 16) at two time points of lactation, Figure S1: Gut microbiota composition as assessed by 16S rDNA sequencing among Kenyan mothers at baseline. (A) All mothers, (B) secretor mothers and (C) non-secretor mothers. The fraction of 16S rDNA reads (%), attributed to specific taxonomic levels is given below the taxon name, Figure S2: Gut microbiota composition as assessed by 16S rDNA sequencing among Kenyan infants at baseline. (A) All infants, (B) infants of secretor mothers and (C) infants of non-secretor mothers. The fraction of 16S rDNA reads (%) attributed to specific taxonomic levels is given below the taxon name.
