# Supplementary material for: Maternal Human Milk Oligosaccharide Profile Modulates the Impact of an Intervention with Iron and Galacto-Oligosaccharides in Kenyan Infants
Source: Nutrients. 2019 Oct 29;11(11):2596. doi: 10.3390/nu11112596 (PMC6893608; doi:10.3390/nu11112596)
Supplement: Supplementary file 1 [file nutrients-11-02596-s001.pdf]

nutrients

Supplementary Material

1. Supplementary Methods

1.1. Laboratory Methods

1.1.1. Breast Milk Samples

Frozen breast milk samples (100 μL) were thawed at 4 °C, diluted 1:50 in water and passed through 0.45 μM filters. Aliquots of 5 to 10 μL were subjected to high-performance anion-exchange chromatography coupled with pulse amperometric detection (HPAE-PAD) to quantitatively determine their HMO composition [1,2]. The HPAE-PAD setup consisted of a Dionex ICS-5000 dual pump system (Thermo Scientific) configurated with a Dionex IonPac NG1 4x35 mm column, used as a trap column to remove hydrophobic contaminants, connected in line to an analytical CarboPac PA1 4x250 mm column equipped with a guard CarboPac PA1 4x50 mm column. The flow rate for the trap column was set at 0.5 mL/min (pump 1) and at 1 mL/min for the analytical column (pump 2). HMOs were separated using a gradient elution program (Table S1) and detected by PAD with the detector set at Carbohydrate Triple Potential position. Authentic HMO standards (Carbosynth, Newbury, UK) were used to calibrate and identify HMO peaks in breast milk samples.

Table S1. Gradient elution program

**Time (min)**	Solvent A, H_2O (%)	Solvent B, 0.5 NaOH (%)	Solvent C, 0.3 M NaOAc (%)
0	80	20	0
10	80	20	0
18	70	20	10
28	70	20	10
32	48	20	32
39	48	20	32
40	0	20	80
45	0	20	80
46	80	20	0
55	80	20	0

1.1.2. Fecal Samples

The following protocol was used for DNA extraction of infant and maternal fecal samples. Samples were first thawed at 4 °C. Then in a 2.0 mL screw-cap tube containing 0.5 g of 0.1 mm sterilized zirconia beads, 250 (± 10%) mg of feces and 700 μL S.T.A.R. buffer (Roche, Indianapolis, IN, USA) were added. The FastPrep instrument (MP Biomedicals, Santa Ana, CA, USA) was used for lysis at 5.5 ms for 3 times 1 min at room temperature. Thereafter samples were incubated, while shaking at 100 rpm and 95 °C for 15 min. The samples were then centrifuged at 16000 g for 5 min at 4 °C. The collected supernatant was kept on ice, while another lysis round as described above, except that only 350 μL S.T.A.R. buffer was added, was done with the remaining stool pellet. The supernatant kept on ice was then pooled with the supernatant from the second lysis round. Purification of DNA was performed on the automated Maxwell instrument (Promega, Madison,

WI, USA) by applying the Maxwell 16 Tissue LEV Total RNA Purification Kit (Promega) according to the manufacturer's instructions. To the first well of the Maxwell cartridge 250 μL of the supernatant was added and finally DNA was eluted with 50 μL of RNAse/DNAse free water.

Using a 2-step PCR, barcoded amplicons from the V3–V4 region of 16S rRNA genes were generated. For initial amplification of the V3–V4 part of the 16S rRNA we used universal primers with the following sequences: forward primer, '5-CCTACGGGAGGCAGCAG-3' (broadly conserved bacterial primer 357F); reverse primer, '5-TACNVGGGTATCTAAKCC' (broadly conserved bacterial primer (with adaptations) 802R) appended with Illumina adaptor sequences. The PCR amplification mixture contained: 1 μL fecal sample DNA, 1 μL barcoded forward primer, 15 μL master mix (1 μL KOD Hot Start DNA Polymerase (1 U/μL; Novagen, Madison, WI, USA), 5 μL KOD-buffer (10×), 3 μL $MgSO_4$ (25 mM), 5 μL dNTP mix (2 mM each)), 1 μL (10 μM) of reverse primer and 33 μL sterile water (total volume 50 μL). PCR conditions were: 95 °C for 2 min followed by 35 cycles of 95 °C for 20 sec, 55 °C for 10 sec, and 70 °C for 15 sec. We then purified the approximately 500 bp PCR amplicons using the MSB Spin PCRapace kit (Invitek, Berlin, Germany). Subsequently we checked concentration and quality with a Qubit fluorometer (Thermo Fisher Scientific). For the second PCR in combination with sample-specific barcoded primers, purified PCR products were shipped to BaseClear BV (Leiden, The Netherlands). PCR products were purified, checked on a Bioanalyzer (Agilent) and quantified. This was followed by multiplexing, clustering and sequencing on an Illumina MiSeq with the paired-end (2x) 300 bp protocol and indexing. We analyzed the sequencing run with the Illumina CASAVA pipeline (v1.8.3) with de-multiplexing based on sample-specific barcodes. From the raw sequencing data we removed the sequence reads of too low quality (only "passing filter" reads were selected) and discarded reads containing adaptor sequences or PhiX control with an in-house filtering protocol. On the remaining reads we performed a quality assessment using the FASTQC quality control tool version 0.10.0.

Selected enteropathogenic bacteria (*Clostridium difficile*, *Clostridium perfringens*, enterohemorrhagic *Escherichia coli* with shiga toxin 1 (EHEC *stx1*), enterohemorrhagic *E. coli* with shiga toxin 2 (EHEC *stx2*), enteropathogenic *E. coli* with the attaching and effacing gene (EPEC *eaeA*), enterotoxigenic *E. coli* with heat-stable enterotoxin (ETEC ST), enterotoxigenic *E. coli* with heat-labile enterotoxin (ETEC LT), *Salmonella* spp. and *Staphylococcus aureus*) were targeted in infant and maternal fecal DNA using quantitative PCR (qPCR). Primers used for qPCR are given in Table S2. Pre-amplification for preparation of the DNA for qPCR was performed using the Fluidigm Preamp Master Mix (Fluidigm, PN 100-5580, California, United States), primers shown in Table S2, and the recommended 12 cycles as described by the manufacturer (Fluidigm Quick Reference for Pre-amplification of cDNA for Gene Expression with Delta Gene Assay, PN 100-5875 C1, Fluidigm). We diluted the final reaction products 5-fold. Sample pre-mix and assay mix were prepared and the qPCR was run with BioMark 96.96 Gene Expression Dynamic Arrays (PN BMK-M-96.69, Fluidigm, South San Francisco, USA) as described by the manufacturer (Advanced Development Protocol 41: Using EvaGreen DNA Binding Dye for Gene Expression with the 48.48 and 96.96 Dynamic Array IFCs, Fluidigm). Target genes were amplified from representative strains to generate standards. Amplicons were then purified, cloned into PGEMT Easy Vector and heterologously expressed in *E. coli* according to supplier instructions (Promega). Concentrations of the plasmids were measured using a Qubit dsDNA BR assay kit (Q32850, Thermo Fisher Scientific) in triplicate on a Spark M10 plate reader (Tecan Group Ltd., Maennedorf, Switzerland). 10-fold dilutions of linearised plasmids harbouring the gene of interest were used to generate standard curves.

Table S2. Primers used for quantitative polymerase chain reaction

Target	Target gene	PCR primer and sequence (5′-3′)	Product size (bp)	Ref.
Clostridium difficile	16S rRNA gene	cdF, TTGAGCGATTTACTTCGGTAAAGA<br>cdR, CCATCCTGTACTGGCTCACCT	157	[3]
Clostridium perfringens	*pcl* (alpha toxin)	plcF, AAGTTACCTTTGCTGCATAATCCC<br>plcR, ATAGATACTCCATATCATCCTGCT	283	[4]
Enterohemorrhagic *Escherichia coli* (EHEC)	*Stx1* (shiga toxin)	JMS1F, GTCACAGTAACAAACCGTAACA<br>JMS1R, TCGTTGACTACTTCTTATCTGGA	95	[5]
Enterohemorrhagic *Escherichia coli* (EHEC)	*Stx2* (shiga toxin)	JMS2F, CGACCCCTCTTGAACATA<br>JMS2G, GATAGACATCAAGCCCTCGT	108	[5]
Enteropathogenic *Escherichia coli* (EPEC)	*eaeA* (*E. coli* attaching and effacing)	EAE-a, ATGCTTAGTGCTGGTTTAGG<br>EAE-b, GCCTTCATCATTTCGCTTTC	248	[5]
Enterotoxigenic *Escherichia coli* (ETEC)	*LT* (heat-labile enterotoxin)	LT-1, AGCAGGTTTCCCACCGGATCACCA<br>LT-2, GTGCTCAGATTCTGGGTCTC	132	[5]
Enterotoxigenic *Escherichia coli* (ETEC)	*ST* (heat-stable enterotoxin)	STa-F, GCTAATGTTGGCAATTTTTATTTCTGTA<br>STa-R, AGGATTACAACAAAGTTCACAGCAGTAA	190	[5]
Salmonella spp.	*invA* (invasion)	invA,139, GTGAAATTATCGCCACGTTCGGGCAA<br>invA, 141, TCATCGCACCGTCAAAGGAACC	284	[5]
Staphylococcus aureus	Nuclease	SA-1, GCGATTGATGGTGATACGGTT<br>SA-2, CAAGCCTTGACGAACTAAAGC	276	[5]

1.2. Data and Statistical Analysis

We defined total fucosylated HMOs as the sum of 2-fucosyllactose (2′FL), 3-fucosyllactose (3′FL), lacto-N-fucopentaose I (LNFPI), lacto-N-fucopentaose II (LNFPII) and lacto-N-fucopentaose III (LNFPIII); total sialylated HMOs as the sum of 6-sialyllactose (6′SL), 3-sialyllactose (3′SL), sialyllacto-N-tetraose d (LSTd), sialyllacto-N-tetraose a (LSTa) and disialyl-lacto-N-teraose (DSLNT); and total non-fucosylated and non-sialylated HMOs as the sum of lacto-N-neotetraose (LNnT), lacto-N-teraose (LNT) and lacto-N-neohexaose (LNnH). We analyzed absolute and relative concentration of HMOs. Relative concentration was calculated by dividing the absolute concentration of the given HMO within the respective sample by the absolute total HMO concentration within the respective sample. We defined a mother as being non-secretor if there were no detectable concentrations of α-1-2-linked fucosylated HMOs (2′FL and LNFPI) in her breast milk sample. Z-scores for length-for-age (LAZ), weight-for-age (WAZ) and weight-for-length (WLZ) were calculated using the WHO Anthro software (Version 3.2.2.1). We calculated body iron stores (BIS) from the sTfR/PF ratio using the following equation: BIS (mg/kg) = ((log(sTfR / PF) − 2.8229) / 0.1207) [6]. We calculated total BIS by multiplying BIS with infant weight.

We analyzed 16S rRNA gene sequences using a workflow based on Qiime 1.8 [7]. We performed operational taxonomic unit (OTU) clustering (open reference), taxonomic assignment and reference alignment with the pick_open_reference_otus.py workflow script of Qiime, using uclust as clustering method (97% identity) and GreenGenes v13.8 as reference database for taxonomic assignment. Reference-based chimera removal was done with Uchime [8]. The RDP classifier version 2.2 was performed for taxonomic classification [9]. For the *Bifidobacterium* genus specific analyses we re-clustered OTUs assigned to *Bifidobacterium* at the 99% identity level and we analyzed these further on composition and diversity. We performed statistical tests as implemented in SciPy (https://www.scipy.org/), downstream of the Qiime-based workflow.

Using the Fluidigm Real-Time PCR Analysis Software we processed qPCR data, including melting curve analysis. For further analyses of the qPCR data we used Excel (Microsoft Office 2010).

Linear regression analysis of the C_T (threshold cycle) values versus the amounts of the template DNA of the standard (log gene copies/μL) was performed to generate the standard curves. For each linear regression we calculated the goodness of fit (r^2) and for each target we calculated primer efficiency. We set the limit of quantification (LOQ) at the last point of the standard line falling in the linear range. We assigned samples below the LOQ the log gene copies/g feces defined as LOQ/2 and defined the sample as below detection limit. We created a summary variable for all assessed virulence and toxin genes (VTGs) of pathogenic *E. coli* (*eaeA*, LT, ST, *stx1*, *stx2*) and for the sum of all pathogens (*C. difficile*, *C. perfringens*, EHEC *stx1*, EHEC *stx2*, EPEC, ETEC LT, ETEC ST, *Samonella* spp. and *S. aureus*). Gene copy number of *C. difficile* was divided by 11 to correct for multiple 16S rDNA genes.

We performed multivariate redundancy analyses (RDAs) on the infant and maternal gut microbiota composition as assessed by 16S rRNA gene sequencing in Canoco version 5.11 using default settings of the analysis type "Constrained" [10]. For the RDA at baseline, we used relative abundance values of OTUs from infant and maternal fecal samples, respectively, as response data, and maternal secretor status as explanatory variable. To investigate the interaction between secretor status and intervention groups in the RDAs, we used relative abundance values of OTUs from infant fecal samples at baseline, week 3 and 4 months, respectively, as response data, and the six intervention–secretor-status groups as explanatory variable. For visualization purposes families (and not OTUs) are plotted as supplementary variables. RDA calculates p values by permutating (Monte Carlo) the sample status.

We tested for between group differences at baseline (secretor vs. non-secretor mothers and infants of secretor mothers vs. infants of non-secretor mothers, respectively) in alpha diversity and abundance of the taxa of primary interest (Bacteroidetes, *Bifidobacterium*, *Clostridiales*, *Enterobacteriaceae* and *Lactobacillus*) and of all taxa, using Wilcoxon rank-sum tests with FDR correction for multiple testing. We tested for differences in alpha diversity (PD whole tree, and for *Bifidobacterium* diversity analysis also Shannon index), phylogenetic distance (weighted UniFrac within individuals between baseline and 3 weeks sample, and between baseline and 4 months) and abundance of the taxa of primary interest and of all taxa, at baseline, 3 weeks and 4 months between the intervention–secretor-status groups (control group infants of secretor mothers (control-S), control group infants of non-secretor mothers (control-NS), Fe group infants of secretor mothers (Fe-S), Fe group infants of non-secretor mothers (Fe-NS), FeGOS group infants of secretor mothers (FeGOS-S), and FeGOS group infants of non-secretor mothers (FeGOS-NS)), using Kruskal-Wallis tests with Dunn's post hoc test to adjust for multiple comparisons. For longitudinal analysis, change of taxon relative abundance over time, 2log ratios were calculated, in which the relative abundance of a taxon at 4 months or 3 weeks was divided by the relative abundance of the same taxon at baseline. Ratios were compared among groups by Wilcoxon rank-sum tests with FDR correction for multiple testing.

We analyzed demographic and anthropometric data, abundance of HMOs, enteropathogen abundances as assessed by qPCR, fecal calprotectin, intestinal fatty acid binding protein (I-FABP), hemoglobin (Hb), plasma ferritin (PF), soluble transferrin receptor (sTfR), body iron stores (BIS), C reactive protein (CRP) and alpha-glycoprotein (AGP) using the R statistical programming environment (R. 3.4.1 software; R Core Team). We run descriptive statistics for all variables and assessed normality by testing the distribution of continuous variables against a normal distribution using the Shapiro-Wilk W test. We transformed the variables with the use of log(x), sqrt(x), or −1 divided by x to correct positive skewness or x^2, x^3, or antilog(x) to correct negative skewness until achieving $W > 0.97$ before proceeding with further data analyses if departing significantly from normality. We show normally distributed data as mean ± SD and non-normally distributed data as median (IQR).

We tested for changes over time of lactation in HMO concentration using Wilcoxon signed-rank tests. We tested for differences in HMO concentrations between secretor and non-secretor mothers using t-tests or Wilcoxon rank-sum tests. Between group differences at baseline (secretor vs. non-secretor mothers and infants of secretor mothers vs. infants of non-secretor mothers, respectively) of enteropathogen abundances as assessed by qPCR, fecal

calprotectin, I-FABP, Hb, PF, sTfR, BIS, CRP, AGP and infant anthropometrics were tested using t-tests or Wilcoxon rank-sum tests. Using linear regression models we investigated if maternal secretor status predicts abundance of enteropathogens, infant anthropometrics, hematological and inflammation status, fecal calprotectin and plasma I-FABP. We investigated the interaction between maternal secretor status (secretor and non-secretor) and intervention groups (control, Fe and FeGOS) on *Bifidobacterium* abundance, enteropathogen abundance, fecal calprotectin, I-FABP, anthropometrics, Hb, PF, sTfR and BIS, by fitting linear mixed-effect models using CRAN package lme. We defined the fixed effects on the variance as time, secretor status, intervention group, time-by-secretor-status, time-by-intervention-group, secretor-status-by-intervention-group and time-by secretor-status-by-intervention-group, and the random structure as the subject to control between-subject differences. We performed model diagnostics by visually inspecting the Tukey-Anscombe plot and the residual plot to verify the assumptions of homoscedasticity and normality of residuals. Post hoc analyses were performed to investigate the effect of time within group (intervention–secretor-status groups: control-S, control-NS, Fe-S, Fe-NS, FeGOS-S, FeGOS-NS) by applying the mvt method. We assessed infant morbidity as longitudinal prevalence, calculated as weeks with illness (RTI or diarrhea and/or mucus in the stool) from baseline to 4 month, divided by the total weeks of observation. We calculated longitudinal prevalence ratio (LPR) and 95 % CI using Poisson regression with robust standard errors [11]. We calculated p values as described by Altman DG and Bland JM [12].

In all analyses, p values < 0.05 were considered statistically significant.

2. Supplementary Results

Table S3. Absolute and relative concentration of human milk oligosaccharides (HMOs) in breast milk samples of Kenyan mothers (n = 75), all and by secretor status.

	**All (n = 75)**		**Secretor (n = 54)**		**Non-Secretor (n = 21)**	
	g/L	*%total*	*g/L*	*%total*	*g/L*	*%total*
2′FL	0.7 (0.0–1.0) [1]	16.8 ± 14.7 [2]	0.8 (0.6–1.2)*	23.4 ± 12.1*	0.0 (0.0–0.0)*	0.0 ± 0.0*
3′FL	0.8 (0.4–1.6)	22.6 ± 18.1	0.8 (0.4–1.3)	20.6 ± 16.0	1.5 (0.4–2.0)	28.0 ± 22.3
LNFPI	0.0 (0.0–0.7)	5.9 ± 9.8	0.0 (0.0–0.7)*	8.2 ± 10.7*	0.0 (0.0–0.0)*	0.0 ± 0.0*
LNFPII	0.0 (0.0–0.8)	8.1 ± 9.7	0.0 (0.0–0.8)°	7.1 ± 9.3	0.8 (0.0–0.8)°	10.8 ± 10.2
LNFPIII	0.5 (0.0–0.6)	7.2 ± 7.8	0.0 (0.0–0.6)*	5.4 ± 6.1 *	0.6 (0.5–0.6)*	11.9 ± 9.6*
LNnT	0.0 (0.0–0.0)	0.8 ± 2.9	0.0 (0.0–0.0)°	1.1 ± 3.4°	0.0 (0.0–0.0)°	0.0 ± 0.0°
LNT	0.5 (0.5–0.7)	13.6 ± 13.4	0.5 (0.5–0.7)°	10.9 ± 7.3*	0.6 (0.5–0.7)°	20.5 ± 21.2*
LNnH	0.0 (0.0–0.0)	0.4 ± 2.5	0.0 (0.0–0.0)	0.6 ± 3.0	0.0 (0.0–0.0)	0.0 ± 0.0
6′SL	0.0 (0.0–0.0)	1.3 ± 4.1	0.0 (0.0–0.0)	1.2 ± 3.7	0.0 (0.0–0.0)	1.6 ± 5.1
3′SL	1.4 (0.0–1.5)	20.4 ± 18.4	1.4 (0.0–1.5)	19.4 ± 17.2	1.4 (0.0–1.4)	22.9 ± 21.3
LSTd	0.0 (0.0–0.0)	1.0 ± 4.5	0.0 (0.0–0.0)	1.4 ± 5.3	0.0 (0.0–0.0)	0.0 ± 0.0
LSTa	0.0 (0.0–0.0)	1.7 ± 5.5	0.0 (0.0–0.0)	0.8 ± 3.0	0.0 (0.0–0.0)	3.9 ± 8.9
DSLNT	0.0 (0.0–0.0)	0.1 ± 0.5	0.0 (0.0–0.0)*	0.0 ± 0.0*	0.0 (0.0–0.0)*	0.2 ± 1.0*
Total HMOs	4.5 (3.1–5.6)	–	4.6 (3.1–5.6)	–	4.0 (3.2–5.6)	–
Fucosylated HMOs	3.0 (1.7–3.7)	60.7 ± 25.4	3.0 (1.9–3.8)	64.6 ± 21.1	2.9 (1.4–3.5)	50.7 ± 32.6
Sialylated HMOs	1.5 (0.0–1.5)	24.5 ± 21.5	1.5 (0.05–1.5)	22.8 ± 19.6	1.5 (0.0–1.5)	28.7 ± 25.9
Non-fucosylated and non-sialylated HMOs	0.5 (0.5–0.7)	14.8 ± 13.9	0.5 (0.5–0.7)	12.6 ± 9.1	0.6 (0.5–0.7)	20.5 ± 21.2

[1] Median (IQR), all such values. [2] Mean ± SD, all such values. 2′FL, 2-fucosyllactose; 3′FL, 3-fucosyllactose; LNFPI, lacto-N-fucopentaose; LNFPII, lacto-N-fucopentaose II; LNFPIII, lacto-N-fucopentaose III; LNnT, lacto-N-neotetraose; LNT, lacto-N-teraose; LNnH, lacto-N-neohexaose; 6′SL, 6-sialyllactose; 3′SL, 3-sialyllactose; LSTd, sialyllacto-N-tetraose d; LSTa, sialyllacto-N-tetraose a; and DSLNT, disialyl-lacto-N-teraose. Absolute concentration given in g/L. Relative concentration given as % of total HMOs (%total). Between group differences (secretor vs. non-secretor) were tested using Wilcoxon rank-sum tests; *p < 0.05, °p < 0.10.

Table S4. Absolute and relative concentration of human milk oligosaccharides (HMOs) in breast milk samples of Kenyan mothers (n = 16) at two time points of lactation.

	**Time 1**		**Time 2**	
	g/L	*%total*	*g/L*	*%total*
2′FL	0.9 (0.0–1.2) [1]	9.9 (0.0–14.3)*	0.5 (0.3–1.2)	21.2 (5.3–28.0)*
3′FL	0.6 (0.4–1.4)	10.9 (6.1–26.2)*	0.8 (0.4–1.4)	23.7 (6.7–45.0)*
LNFPI	0.7 (0.0–0.8)	6.4 (0.0–10.9)	0.0 (0.0–0.8)	0.0 (0.0–15.1)
LNFPII	0.4 (0.0–0.8)	3.2 (0.0–11.7)	0.0 (0.0–0.8)	0.0 (0.0–14.5)
LNFPIII	0.5 (0.0–0.6)	5.8 (0.0–8.9)	0.5 (0.0–0.6)	8.6 (0.0–11.5)
LNnT	0.0 (0.0–0.5)*	0.0 (0.0–5.4)*	0.0 (0.0–0.0)*	0.0 (0.0–0.0)*
LNT	0.6 (0.5–0.8)	10.0 (7.9–11.3)*	0.6 (0.5–0.7)	11.4 (9.9–21.4)*
LNnH	Not detected	Not detected	Not detected	Not detected
6′SL	1.1 (0.8–1.1)*	13.1 (6.7–16.2)*	0.0 (0.0–0.0)*	0.0 (0.0–0.0)*
3′SL	1.4 (1.4–1.4)	21.4 (15.4–26.7)	1.5 (0.0–1.5)	20.3 (0.0–28.1)
LSTd	Not detected	Not detected	Not detected	Not detected
LSTa	1.0 (0.0–1.0)*	9.3 (0.0–14.6)*	0.0 (0.0–0.0)*	0.0 (0.0–0.0)*
DSLNT	Not detected	Not detected	Not detected	Not detected
Total HMOs	6.6 (5.2–7.7)*	–	4.9 (2.4–5.4)*	–
Fucosylated HMOs	2.8 (2.5–3.3)	46.4 (38.7–55.4)*	3.1 (1.8–3.6)	68.4 (61.0–75.8)*
Sialylated HMOs	3.5 (2.5–3.5)*	37.6 (31.4–49.1)*	1.5 (0.0–1.5)*	21.6 (0.0–28.5)*
Non fucosylated and non sialylated HMOs	0.7 (0.5–1.2)	12.5 (9.4–15.5)	0.6 (0.5–0.7)	11.4 (9.9–21.4)

[1]Median (IQR), all such values. 2′FL, 2-fucosyllactose; 3′FL, 3-fucosyllactose; LNFPI, lacto-N-fucopentaose; LNFPII, lacto-N-fucopentaose II; LNFPIII, lacto-N-fucopentaose III; LNnT, lacto-N-neotetraose; LNT, lacto-N-teraose; LNnH, lacto-N-neohexaose; 6′SL, 6-sialyllactose; 3′SL, 3-sialyllactose; LSTd, sialyllacto-N-tetraose d; LSTa, sialyllacto-N-tetraose a; and DSLNT, disialyl-lacto-N-teraose. Absolute concentration given in g/L. Relative concentration given as % of total HMOs (%total). Breast milk samples at time 2 were collected 3 months later in lactation than breast milk samples at time 1. Between group differences (secretor vs. non-secretor) were tested using Wilcoxon rank-sum test; *$p < 0.05$, °$p < 0.10$.

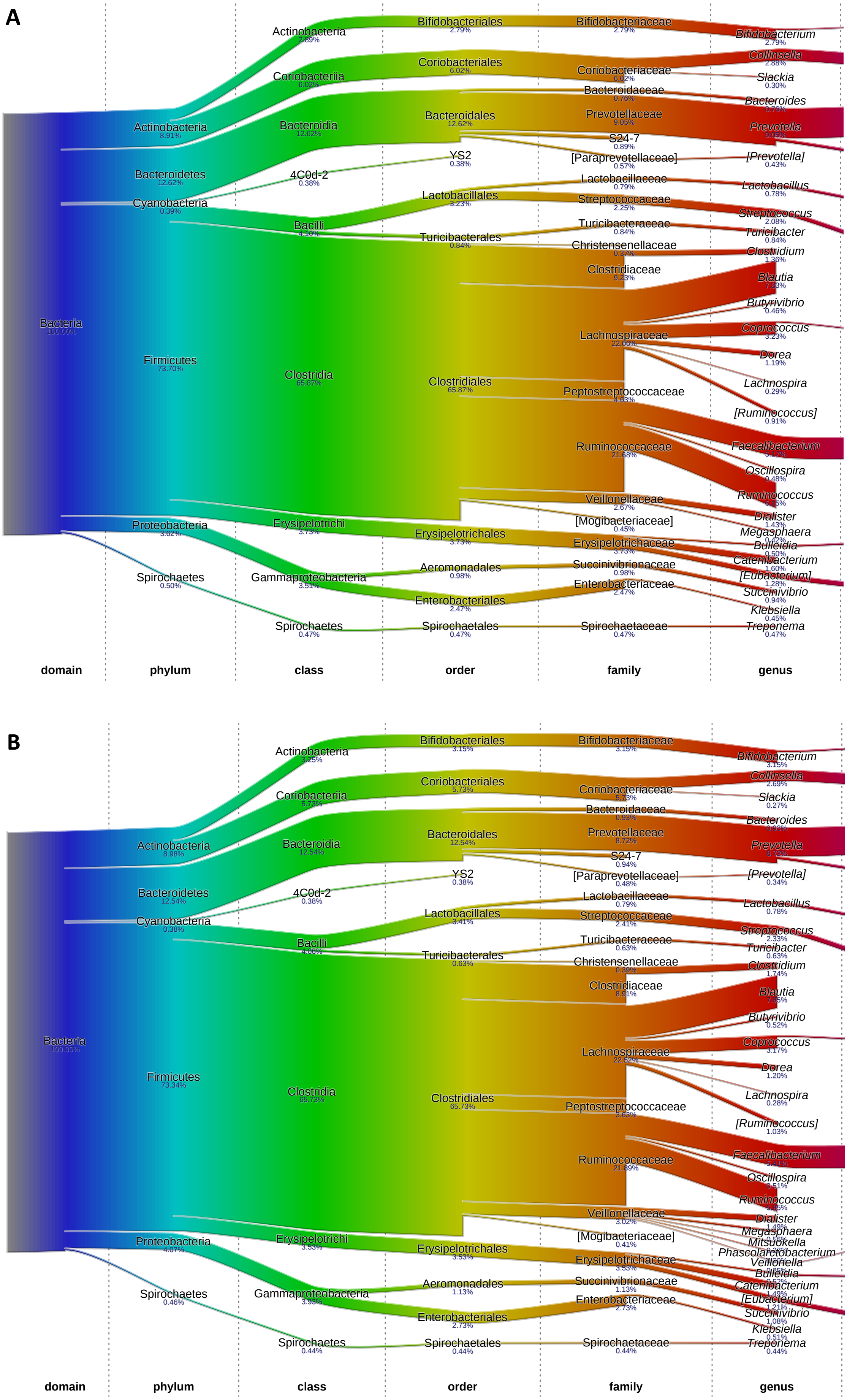
A
Bacteria
100.00%
Actinobacteria
8.91%
Bacteroidetes
12.62%
Cyanobacteria
0.39%
Firmicutes
73.70%
Proteobacteria
3.62%
Spirochaetes
0.50%
Actinobacteria
2.89%
Coriobacteriia
6.02%
Bacteroidia
12.62%
4C0d-2
0.38%
Bacilli
4.10%
Clostridia
65.87%
Erysipelotrichi
3.73%
Gammaproteobacteria
3.51%
Spirochaetes
0.47%
Bifidobacteriales
2.79%
Coriobacteriales
6.02%
Bacteroidales
12.62%
YS2
0.38%
Lactobacillales
3.23%
Turicibacterales
0.84%
Clostridiales
65.87%
Erysipelotrichales
3.73%
Aeromonadales
0.98%
Enterobacteriales
2.47%
Spirochaetales
0.47%
Bifidobacteriaceae
2.79%
Coriobacteriaceae
6.02%
Bacteroidaceae
0.76%
Prevotellaceae
9.05%
S24-7
0.89%
[Paraprevotellaceae]
0.57%
Lactobacillaceae
0.79%
Streptococcaceae
2.25%
Turicibacteraceae
0.84%
Christensenellaceae
0.37%
Clostridiaceae
9.23%
Lachnospiraceae
22.66%
Peptostreptococcaceae
4.03%
Ruminococcaceae
21.58%
Veillonellaceae
2.67%
[Mogibacteriaceae]
0.45%
Erysipelotrichaceae
3.73%
Succinivibrionaceae
0.98%
Enterobacteriaceae
2.47%
Spirochaetaceae
0.47%
Bifidobacterium
2.79%
Collinsella
2.88%
Slackia
0.30%
Bacteroides
0.76%
Prevotella
9.05%
[Prevotella]
0.43%
Lactobacillus
0.78%
Streptococcus
2.08%
Turicibacter
0.84%
Clostridium
1.36%
Blautia
7.83%
Butyrivibrio
0.46%
Coprococcus
3.23%
Dorea
1.19%
Lachnospira
0.29%
[Ruminococcus]
0.91%
Faecalibacterium
Oscillospira
0.48%
Ruminococcus
Dialister
1.43%
Megasphaera
0.42%
Bulleidia
0.50%
Catenibacterium
1.60%
[Eubacterium]
1.28%
Succinivibrio
0.94%
Klebsiella
0.45%
Treponema
0.47%
domain
phylum
class
order
family
genus
B
Bacteria
100.00%
Actinobacteria
8.98%
Bacteroidetes
12.54%
Cyanobacteria
0.38%
Firmicutes
73.34%
Proteobacteria
4.07%
Spirochaetes
0.46%
Actinobacteria
3.25%
Coriobacteriia
5.73%
Bacteroidia
12.54%
4C0d-2
0.38%
Bacilli
4.06%
Clostridia
65.73%
Erysipelotrichi
3.53%
Gammaproteobacteria
3.93%
Spirochaetes
0.44%
Bifidobacteriales
3.15%
Coriobacteriales
5.73%
Bacteroidales
12.54%
YS2
0.38%
Lactobacillales
3.41%
Turicibacterales
0.63%
Clostridiales
65.73%
Erysipelotrichales
3.53%
Aeromonadales
1.13%
Enterobacteriales
2.73%
Spirochaetales
0.44%
Bifidobacteriaceae
3.15%
Coriobacteriaceae
5.73%
Bacteroidaceae
0.93%
Prevotellaceae
8.72%
S24-7
0.94%
[Paraprevotellaceae]
0.48%
Lactobacillaceae
0.79%
Streptococcaceae
2.41%
Turicibacteraceae
0.63%
Christensenellaceae
0.39%
Clostridiaceae
8.91%
Lachnospiraceae
22.52%
Peptostreptococcaceae
3.63%
Ruminococcaceae
21.89%
Veillonellaceae
3.02%
[Mogibacteriaceae]
0.41%
Erysipelotrichaceae
3.53%
Succinivibrionaceae
1.13%
Enterobacteriaceae
2.73%
Spirochaetaceae
0.44%
Bifidobacterium
3.15%
Collinsella
2.69%
Slackia
0.27%
Bacteroides
0.93%
Prevotella
8.72%
[Prevotella]
0.34%
Lactobacillus
0.78%
Streptococcus
2.33%
Turicibacter
0.63%
Clostridium
1.74%
Blautia
7.65%
Butyrivibrio
0.52%
Coprococcus
3.17%
Dorea
1.20%
Lachnospira
0.28%
[Ruminococcus]
1.03%
Faecalibacterium
Oscillospira
0.51%
Ruminococcus
Dialister
Megasphaera
Mitsuokella
Phascolarctobacterium
Veillonella
Bulleidia
Catenibacterium
[Eubacterium]
1.21%
Succinivibrio
1.08%
Klebsiella
0.51%
Treponema
0.44%
domain
phylum
class
order
family
genus

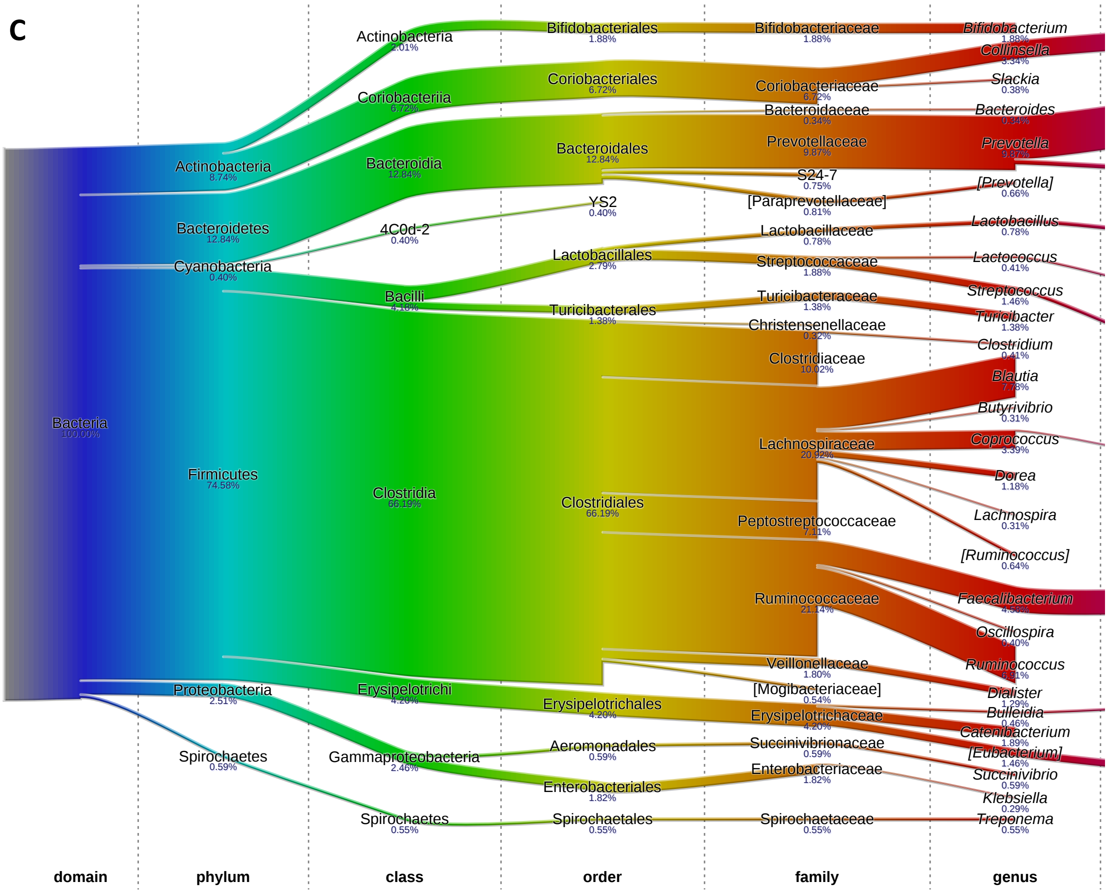

Figure S1. Gut microbiota composition as assessed by 16S rDNA sequencing among Kenyan mothers at baseline. **A)** All mothers, **B)** secretor mothers and **C)** non-secretor mothers. The fraction of 16S rDNA reads (%) attributed to specific taxonomic levels is given below the taxon name.

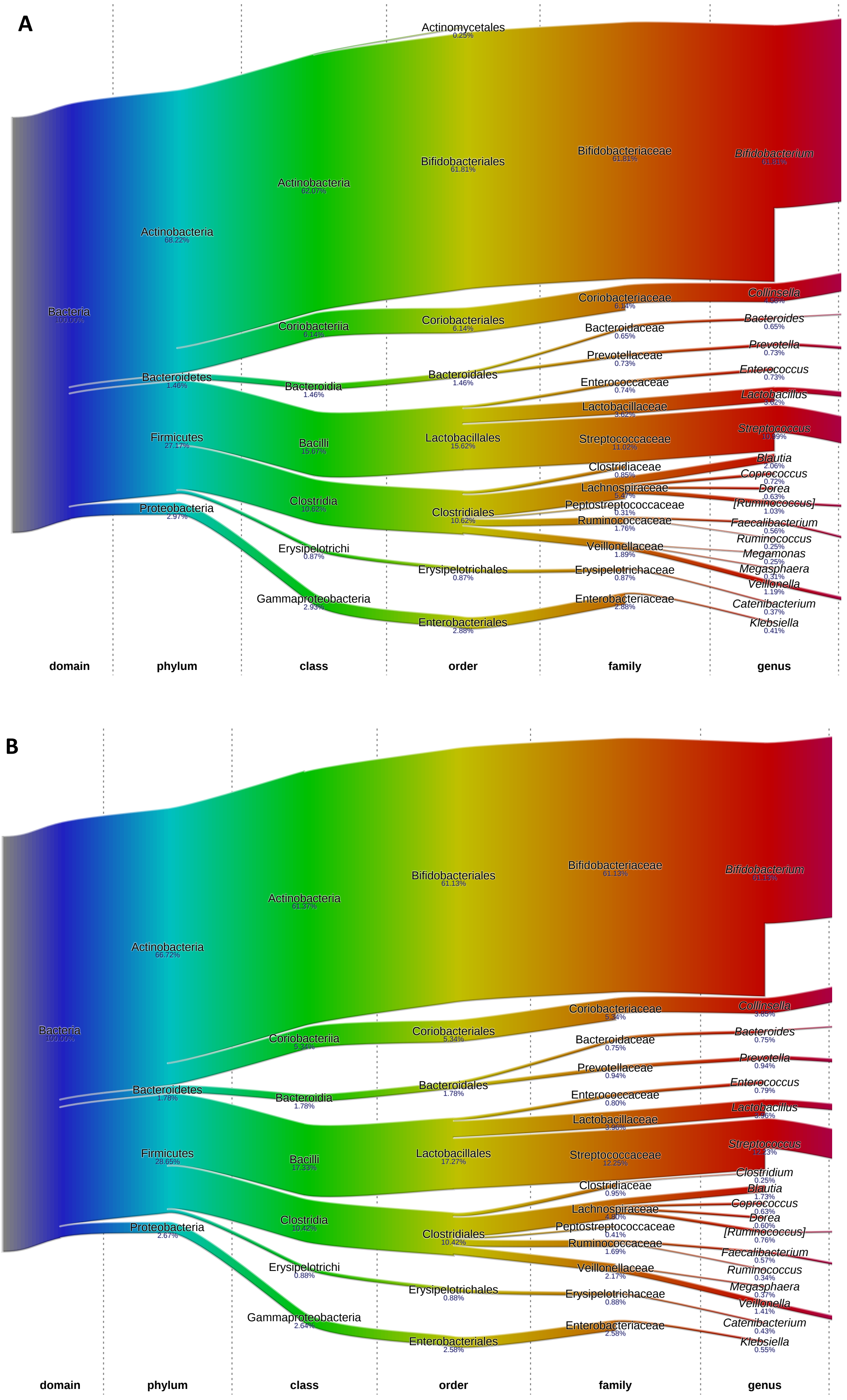
A
Actinomycetales
0.25%
Bacteria
100.00%
Actinobacteria
68.22%
Actinobacteria
62.07%
Bifidobacteriales
61.81%
Bifidobacteriaceae
61.81%
Bifidobacterium
61.81%
Coriobacteriia
6.14%
Coriobacteriales
6.14%
Coriobacteriaceae
6.14%
Collinsella
Bacteroidetes
1.46%
Bacteroidia
1.46%
Bacteroidales
1.46%
Bacteroidaceae
0.65%
Bacteroides
0.65%
Prevotellaceae
0.73%
Prevotella
0.73%
Enterococcaceae
0.74%
Enterococcus
0.73%
Lactobacillaceae
Lactobacillus
Firmicutes
27.17%
Bacilli
15.67%
Lactobacillales
15.62%
Streptococcaceae
11.02%
Streptococcus
10.99%
Clostridiaceae
0.85%
Blautia
2.06%
Coprococcus
0.72%
Lachnospiraceae
Dorea
0.63%
Peptostreptococcaceae
0.31%
[Ruminococcus]
1.03%
Clostridia
10.62%
Clostridiales
10.62%
Ruminococcaceae
1.76%
Faecalibacterium
0.56%
Ruminococcus
0.25%
Veillonellaceae
1.89%
Megamonas
0.25%
Megasphaera
0.31%
Proteobacteria
2.97%
Erysipelotrichi
0.87%
Erysipelotrichales
0.87%
Erysipelotrichaceae
0.87%
Veillonella
1.19%
Gammaproteobacteria
2.93%
Enterobacteriaceae
2.88%
Catenibacterium
0.37%
Enterobacteriales
2.88%
Klebsiella
0.41%
domain
phylum
class
order
family
genus
B
Bacteria
100.00%
Actinobacteria
66.72%
Actinobacteria
61.37%
Bifidobacteriales
61.13%
Bifidobacteriaceae
61.13%
Bifidobacterium
61.13%
Coriobacteriia
5.34%
Coriobacteriales
5.34%
Coriobacteriaceae
5.34%
Collinsella
Bacteroidaceae
0.75%
Bacteroides
0.75%
Prevotellaceae
0.94%
Prevotella
0.94%
Bacteroidetes
1.78%
Bacteroidia
1.78%
Bacteroidales
1.78%
Enterococcaceae
0.80%
Enterococcus
0.79%
Lactobacillaceae
Lactobacillus
Firmicutes
28.65%
Bacilli
17.33%
Lactobacillales
17.27%
Streptococcaceae
12.25%
Streptococcus
12.23%
Clostridium
0.25%
Clostridiaceae
0.95%
Blautia
1.73%
Coprococcus
0.63%
Lachnospiraceae
4.80%
Dorea
0.60%
Clostridia
10.42%
Clostridiales
10.42%
Peptostreptococcaceae
0.41%
[Ruminococcus]
0.76%
Proteobacteria
2.67%
Ruminococcaceae
1.69%
Faecalibacterium
0.57%
Ruminococcus
0.34%
Erysipelotrichi
0.88%
Veillonellaceae
2.17%
Megasphaera
0.37%
Erysipelotrichales
0.88%
Erysipelotrichaceae
0.88%
Veillonella
1.41%
Gammaproteobacteria
2.64%
Enterobacteriaceae
2.58%
Catenibacterium
0.43%
Enterobacteriales
2.58%
Klebsiella
0.55%
domain
phylum
class
order
family
genus

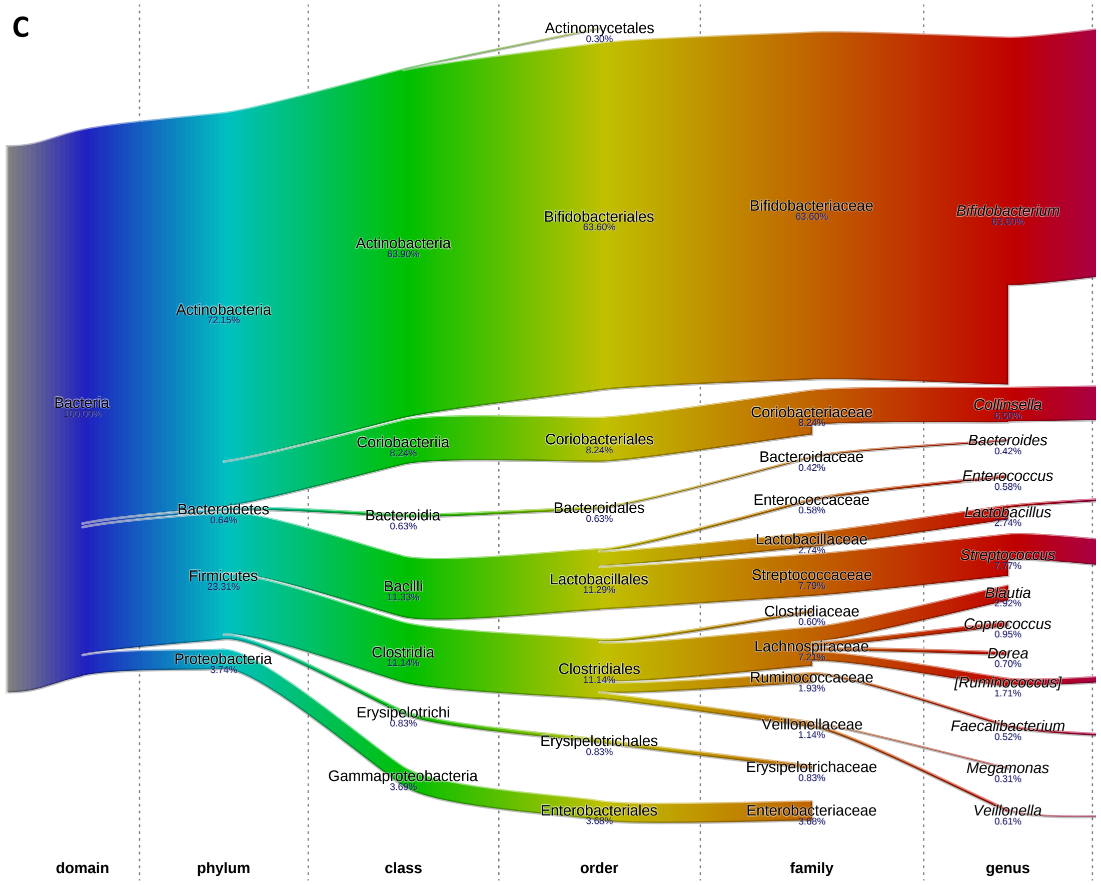

Figure S2. Gut microbiota composition as assessed by 16S rDNA sequencing among Kenyan infants at baseline. **A)** All infants, **B)** infants of secretor mothers and **C)** infants of non-secretor mothers. The fraction of 16S rDNA reads (%) attributed to specific taxonomic levels is given below the taxon name.

4. References

1. Thurl, S.; Muller-Werner, B.; Sawatzki, G. Quantification of individual oligosaccharide compounds from human milk using high-ph anion-exchange chromatography. *Analytical biochemistry* **1996**, *235*, 202-206.
2. Rudloff, S.; Pohlentz, G.; Diekmann, L.; Egge, H.; Kunz, C. Urinary excretion of lactose and oligosaccharides in preterm infants fed human milk or infant formula. *Acta paediatrica (Oslo, Norway : 1992)* **1996**, *85*, 598-603.
3. Rinttila, T.; Kassinen, A.; Malinen, E.; Krogius, L.; Palva, A. Development of an extensive set of 16s rdna-targeted primers for quantification of pathogenic and indigenous bacteria in faecal samples by real-time pcr. *J Appl Microbiol* **2004**, *97*, 1166-1177.
4. Rinttila, T.; Lyra, A.; Krogius-Kurikka, L.; Palva, A. Real-time pcr analysis of enteric pathogens from fecal samples of irritable bowel syndrome subjects. *Gut Pathog* **2011**, *3*, 6.
5. Fukushima, H.; Tsunomori, Y.; Seki, R. Duplex real-time sybr green pcr assays for detection of 17 species of food- or waterborne pathogens in stools. *Journal of Clinical Microbiology* **2003**, *41*, 5134-5146.
6. Cook, J.D.; Flowers, C.H.; Skikne, B.S. The quantitative assessment of body iron. *Blood* **2003**, *101*, 3359-3364.
7. Caporaso, J.G.; Kuczynski, J.; Stombaugh, J.; Bittinger, K.; Bushman, F.D.; Costello, E.K.; Fierer, N.; Pena, A.G.; Goodrich, J.K.; Gordon, J.I., *et al.* Qiime allows analysis of high-throughput community sequencing data. *Nature methods* **2010**, *7*, 335-336.
8. Edgar, R.C.; Haas, B.J.; Clemente, J.C.; Quince, C.; Knight, R. Uchime improves sensitivity and speed of chimera detection. *Bioinformatics* **2011**, *27*, 2194-2200.
9. Cole, J.R.; Wang, Q.; Cardenas, E.; Fish, J.; Chai, B.; Farris, R.J.; Kulam-Syed-Mohideen, A.S.; McGarrell, D.M.; Marsh, T.; Garrity, G.M., *et al.* The ribosomal database project: Improved alignments and new tools for rrna analysis. *Nucleic acids research* **2009**, *37*, D141-145.
10. Braak, C.J.F.; Smilauer, P. Canoco reference manual anc canodraw for windows user's guide: Software for canonical communiy ordination. *Microcomputer Power (Ithaca, NY, USA)* **2002**.
11. Zou, G. A modified poisson regression approach to prospective studies with binary data. *American Journal of Epidemiology* **2004**, *159*, 702-706.
12. Altman, D.G.; Bland, J.M. How to obtain the p value from a confidence interval. *Bmj* **2011**, *343*, d2304-d2304.
